# Impact of the COVID-19 pandemic on CVD prevention between different socioeconomic groups in Switzerland

**DOI:** 10.1136/openhrt-2023-002368

**Published:** 2023-09-19

**Authors:** Ko Ko Maung, Pedro Marques-Vidal

**Affiliations:** Department of Medicine, Internal Medicine, Centre Hospitalier Universitaire Vaudois, Lausanne, Switzerland

**Keywords:** hypertension, diabetes mellitus, hyperlipidemias, epidemiology

## Abstract

**Background:**

The COVID-19 pandemic disrupted the continuing management of cardiovascular disease (CVD) risk factors in the population. Socioeconomic status (SES) is a major determinant of health. Whether the COVID-19 pandemic increased, the SES gap in CVD risk factors is unknown.

**Aims:**

To compare the management of CVD risk factors and the SES gap before and during the pandemic.

**Methods:**

Cross-sectional study conducted between 2018 and 2021 in Lausanne, Switzerland. Prevalence, awareness, treatment and control rates of hypertension, dyslipidaemia and diabetes were compared between the periods before (N=2416, 45.2% men, 65.3±9.8 years) and during (N=776, 44.5% men, 63.9±9.1 years) the COVID-19 pandemic. SES was defined by education and categorised as low (compulsory or apprenticeship), middle (high school) and high (university).

**Results:**

After multivariable analysis, the prevalence of hypertension increased, and awareness decreased during the pandemic: OR and (95% CI) 1.26 (1.04 to 1.53) and 0.70 (0.53 to 0.94), respectively. For dyslipidaemia, prevalence decreased during the pandemic 0.82 (95% CI 0.69 to 0.98); awareness did not change. For diabetes, prevalence did not change but awareness increased 5.76 (95% CI 1.23 to 27.04). No differences were found before and during the pandemic regarding treatment and control for all CVD risk factors. Relative to high SES, a decrease in hypertension awareness among middle SES categories was observed during the pandemic (OR and 95% CI 1.11 (0.73 to 1.69) before and 0.45 (95% CI 0.23 to 0.85) during, p for interaction<0.05), while no other changes were found.

**Conclusion:**

Prevalence and management of CVD risk factors changed little during the pandemic. The SES gap did not increase except for hypertension awareness.

**WHAT IS ALREADY KNOWN ON THIS TOPIC**

- The COVID-19 pandemic and the restrictive measures posed an inverse impact on the cardiovascular disease (CVD) services of most countries. Additionally, management of CVD risk factors shows a socioeconomic gradient; subjects with a higher socioeconomic status (SES) present with higher control levels than subjects of lower SES. So far, little is known if the COVID-19 pandemic affected the management of CVD risk factors, and if it widened the SES gap in CVD prevention.

**WHAT THIS STUDY ADDS**

- Prevalence and management of CVD risk factors in Switzerland changed little during the pandemic. Regarding the CVD risk factors management between different SES, the SES gap did not increase except for hypertension awareness.

**HOW THIS STUDY MIGHT AFFECT RESEARCH, PRACTICE OR POLICY**

- We expect this analysis will be of great help in better understanding the impacts of the pandemic on CVD prevention, and in implementing strategies and planning to allow maintaining adequate health provision to patients with CVD in future COVID-19 outbreaks or similar extreme events.

## Introduction

The burden of cardiovascular diseases (CVD) had been on the rise in Switzerland with the ageing population^1^ and escalating multimorbidity.^2^ CVD is caused by major risk factors such as hypertension, dyslipidaemia, smoking and diabetes; European guidelines regarding the prevention of CVD by tackling those risk factors have been issued.^3^ Still, management of CVD risk factors is far from optimal in Europe^4^ and Switzerland is no exception.^5–7^ Management of CVD risk factors also shows a socioeconomic gradient, subjects with a higher socioeconomic status (SES) presenting with higher control levels than subjects of lower SES.^8^ This leads to a considerable loss of opportunity among the lowest SES groups, who present with higher CVD rates, and earlier CVD events than higher SES groups.^9^

The COVID-19 pandemic posed a substantial global healthcare crisis. As of 11 October 2022, there have been over 630 million confirmed cases of COVID-19, and over 6.5 million deaths worldwide.^10^ As a response to the unprecedented rapidly spreading pandemic, containment and mitigation measures were done, leading to an inverse impact on the CVD services of most countries.^11^

In addition to exacerbating underlying CVD through direct and indirect effects, the COVID-19 crisis has also reinforced social inequalities.^12^ Emerging evidence suggests that the combined effect of COVID-19 and pre-existing inequalities in CVD and the social determinants of health might cause a ‘syndemic effect’, that is, a co-occurring synergistic pandemic exacerbating social and economic inequalities, particularly to the disadvantaged groups.^13^ As CVD is strongly associated with health inequalities and its risk factors are highly prevalent among socioeconomic disadvantaged groups,^14^ it is likely that the COVID-19 crisis has reinforced the previously existing social inequalities. Indeed, subjects with low SES are more frequently inadequately managed for their CVD and contaminated by COVID-19.^15^ So far, little is known if the COVID-19 pandemic affected the management of CVD risk factors, and if it widened the SES gap in CVD prevention. Such information is vital if one wants to reduce the SES gap in health by promoting equity.

Hence, we aimed to assess the effect of the COVID-19 pandemic on the socialeconomic gap regarding management of cardiovascular risk factors in the general population. Our initial hypothesis is that the socioeconomic gap increased during the pandemic.

## Methods

### Study setting

The CoLaus|PsyCoLaus study (https://www.colaus-psycolaus.ch/) is a population-based study investigating the epidemiology and genetic determinants of psychiatric and CVD in Lausanne, Switzerland.^16^ The study is mostly composed of Caucasian participants (92.6%). For this study, only data from the third follow-up was considered. Two study periods were defined: before and during the pandemic; the latter period was defined as starting after 1 March 2020.

No patient or public was involved in the drafting and conduction of the CoLaus|PsyColaus study as at that time it was not considered necessary by the investigators or the ethics committee.

### Cardiovascular risk factors

In each survey, participants attended the CoLaus survey in the morning after an overnight fast and answered questionnaires, underwent a clinical examination and blood samples were drawn for analyses. Biological assays were conducted on fresh blood samples by the Clinical Laboratory of the Lausanne University Hospital (CHUV) on a Cobas 8000 (Roche Diagnostics, Basel, Switzerland).

Blood pressure (BP) was measured using an Omron HEM-907 automated oscillometric sphygmomanometer after at least a 10 min rest in a seated position, and the average of the last two measurements was used. Hypertension was defined by a systolic blood pressure (SBP) ≥ 140 mm Hg or a diastolic blood pressure (DBP) ≥ 90 mm Hg or presence of antihypertensive drug treatment or a positive answer to the question ‘Did a doctor tell you that you were hypertensive?’. Awareness of hypertension was defined if the participant replied positively to the question ‘Did a doctor tell you that you were hypertensive?’ or if they reported any antihypertensive drug treatment. Participants with hypertension were considered as being treated if they reported any antihypertensive drug treatment. Participants treated for hypertension were considered as controlled if they had both SBP<140 mm Hg and DBP<90 mm Hg.

Diabetes was defined as a glycated haemoglobin (HbA_1_c) ≥ 6.5% (48 mmol/mol) or presence of antidiabetic drug treatment or a positive answer to the question ‘Have you ever been told that you had diabetes?’. Awareness of diabetes was considered if participants replied positively to the question ‘Have you ever been told that you had diabetes?’ or if they reported any antidiabetic drug treatment. Participants with diabetes were considered as being treated if they reported any antidiabetic drug treatment. Participants treated for diabetes were considered as controlled if their HbA_1_c was<6.5%.

Dyslipidaemia was defined as a total cholesterol ≥6.18 mmol/L or a low-density lipoprotein (LDL) cholesterol ≥4.1 mmol/L or triglycerides ≥5.6 mmol/L or a high-density lipoprotein (HDL) cholesterol <1 mmol/L in male and <1.3 mmol/L in female^17^ or presence of antilipid drug treatment or a positive answer to the question ‘Have you ever been told that you had high cholesterol?’. Awareness of dyslipidaemia was considered if the participant replied positively to the question ‘Have you ever been told that you had high cholesterol?’ or if they reported any type of hypolipidemic drug treatment. Participants with dyslipidaemia were considered as treated if they reported any type of hypolipidemic drug treatment. Participants treated for dyslipidaemia were considered as controlled if their total cholesterol level is <5.18 mmol/L and LDL cholesterol level is <1.8 mmol/L and triglycerides level is <1.7 mmol/L or HDL cholesterol level is ≥1.5 mmol/L.^17^

### Socioeconomic status

SES was defined by self-reported educational level and categorised as low (mandatory or apprenticeship), middle (high school) and high (university).

### Covariates

Other data were collected by questionnaire and included gender, age, marital status (alone/couple), personal and family history of CVD, smoking (never/former/current) and alcohol consumption (yes/no).

Body weight and height were measured with participants barefoot and in light indoor clothes. Body mass index (BMI) was computed and categorised as normal, overweight (25–29.9 kg/m^2^) and obese (≥30 kg/m^2^).

### Inclusion and exclusion criteria

Participants were considered as eligible if they participated in the third follow-up. Participants were excluded if they (1) missed any data for hypertension, dyslipidaemia or diabetes and (2) missed any covariate.

### Statistical analysis

Statistical analyses were conducted using Stata V.16 for Windows (StataCorp). Participant characteristics were expressed as a number (percentage) for categorical variables or as an average±SD for continuous variables. Between-group comparisons were performed using a χ^2^ or Fisher’s exact test for categorical variables and a student’s t-test or Kruskal-Wallis test for continuous variables.

Multivariable analyses were conducted using logistic regression for categorical outcomes, and results were expressed as OR and (95% CI). Analyses were adjusted on gender, age (continuous), education (high, middle, low), occupation (yes, no), smoking (never, former, current), BMI categories (normal, overweight, obese) and alcohol consumption (yes, no). Each CVD risk factor was also adjusted for the other two risk factors. Due to the small number of non-Caucasian participants, it was not possible to consider ethnicity in the multivariable models. Changes in the association between SES and management of CVD risk factors induced by the pandemic were assessed by including an interaction term between educational level or occupation and period. Statistical significance was considered for a two-sided test with p<0.05.

## Results

### Characteristics of the participants

Out of the 3751 participants who attended the CoLaus study during the study period, 559 (14.9%) were excluded. The reasons for exclusion are indicated in online supplemental figure 1 and the bivariate analysis of the general characteristics between the included and excluded participants is summarised in online supplemental table 1. Compared with the included participants, the excluded participants were older, less frequently Swiss-born, less educated, more obese, more frequently current smokers, but less frequently drinkers.

10.1136/openhrt-2023-002368.supp1 Supplementary data

10.1136/openhrt-2023-002368.supp2 Supplementary data

Table 1 presents the general characteristics of the included participants who attended the study before and during the pandemic. Out of the 3192 participants, 2416 (76%) attended before and 776 (24%) attended during the pandemic. Compared with participants who attended before the pandemic, participants who attended during the pandemic were younger, more highly educated, less obese, less frequently drinkers, but slightly more current smokers. There were no significant differences in gender ratio, country of birth and history of CVD.

**Table 1:** Characteristics of participants attending the CoLaus study before and during the COVID-19 pandemic, Lausanne, Switzerland

	Before	During	P value
Sample size	2416	776	
Women (%)	1324 (54.8)	431 (55.5)	0.719
Age (years)	65.3±9.8	63.9±9.1	<0.001
Swiss born (%)	1568 (64.9)	512 (66.0)	0.583
Education (%)			
Low	522 (21.6)	211 (27.2)	<0.001
Middle	702 (29.1)	196 (25.3)	
High	1192 (49.3)	369 (47.6)	
Occupational status			
Not working	1251 (52.0)	383 (49.4)	0.208
Working	1154 (48.0)	392 (50.6)	
Smoking categories (%)			
Never	1060 (43.9)	348 (44.9)	<0.001
Former	976 (40.4)	300 (38.7)	
Current	380 (15.7)	128 (16.5)	
Body mass index (BMI) (kg/m^2^)	26.3±4.6	26.3±4.9	0.834
BMI categories (%)			
Normal	1015 (42.0)	349 (45.0)	<0.001
Overweight	941 (39.0)	282 (36.3)	
Obese	460 (19.0)	145 (18.7)	
History of CVD (%)			
Personal	312 (12.9)	113 (14.6)	0.240
Family	1734 (71.8)	536 (69.1)	0.149
Alcohol drinker (%)	1712 (70.9)	277 (35.7)	<0.001

Results are expressed as number of participants (percentage) for categorical variables and as average±SD for continuous variables. Between-group comparisons performed using χ^2^ for categorical variables and Student’s t-test for continuous variables.

CVD, cardiovascular disease.

### Prevalence and management of CVD risk factors before and during the COVID-19 pandemic

The results of the bivariate analysis of prevalence, treatment and control rates for CVD risk factors are provided in table 2. For hypertension and diabetes, prevalence did not change, while awareness decreased; no changes were found for treatment and control. For dyslipidaemia, prevalence decreased, while no changes were found for awareness, treatment and control. Moreover, no gender-specific differences regarding prevalence, awareness, control and treatment of the CVD risk factors were seen before and during the pandemic (online supplemental table 2).

**Table 2:** Bivariate analysis of the management of cardiovascular risk factors before and during the COVID-19 pandemic, CoLaus study, Lausanne, Switzerland

	Before	During	P value
Sample size	2416	776	
Hypertension (%)			
Prevalence	1259 (52.1)	404 (52.1)	0.981
Awareness	1009 (80.1)	299 (74.0)	<0.05
Treatment	861 (85.3)	260 (87.0)	0.481
Control	519 (60.3)	161 (61.9)	0.634
Dyslipidaemia (%)			
Prevalence	1169 (48.4)	328 (42.3)	<0.05
Awareness	732 (62.6)	209 (63.7)	0.715
Treatment	407 (55.6)	115 (55.0)	0.882
Control	88 (21.6)	31 (27.0)	0.229
Diabetes (%)			
Prevalence	240 (9.9)	78 (10.1)	0.924
Awareness	213 (88.8)	76 (97.4)	<0.05
Treatment	168 (78.9)	65 (85.5)	0.208
Control	62 (36.9)	32 (49.2)	0.085

Results are expressed as number of participants (percentage). Denominator for prevalence is total sample size; denominator for awareness is the number of participants with the condition (hypertension, diabetes or dyslipidaemia); denominator for treatment is the number of participants aware of the condition; denominator for control is the number of participants treated for the condition. Between-group comparisons performed using χ^2^.

**Figure 1:**
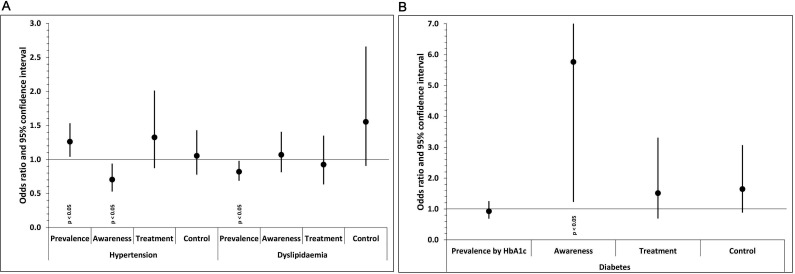
OR of prevalence, awareness, treatment and control of cardiovascular disease risk factors between educational statues during (2020–2021) the pandemic. (Compared with prepandemic period; 2018–2019). (A) prevalence, awareness, treatment and control of hypertensive and dyslipidaemia participants during the pandemic; (B) prevalence, awareness, treatment and control of diabetic participants during the pandemic.HbA1c, glycated haemoglobin.

The results of the multivariate analysis adjusting for gender, age, education status, alcohol consumption, smoking status, BMI categories and the presence of other CVD risk factors are provided in table 3 and figure 1. Prevalence of hypertension increased, and awareness decreased during the pandemic. For diabetes, prevalence did not change but awareness increased. For dyslipidaemia, prevalence decreased during the pandemic, but awareness did not change. No differences were found before and during the pandemic regarding treatment and control for all CVD risk factors.

**Table 3:** Multivariable analysis of the management of cardiovascular risk factors during the COVID-19 pandemic, CoLaus study, Lausanne, Switzerland

	During the pandemic	P value
Hypertension		
Prevalence	1.26 (1.04 to 1.53)	<0.05
Awareness	0.70 (0.53 to 0.94)	<0.05
Treatment	1.32 (0.87 to 2.01)	0.187
Control	1.05 (0.78 to 1.43)	0.738
Dyslipidaemia		
Prevalence	0.82 (0.69 to 0.98)	<0.05
Awareness	1.07 (0.81 to 1.41)	0.633
Treatment	0.93 (0.63 to 1.35)	0.686
Control	1.55 (0.91 to 2.66)	0.109
Diabetes		
Prevalence	0.93 (0.69 to 1.26)	0.630
Awareness	5.76 (1.23 to 27.04)	<0.05
Treatment	1.51 (0.69 to 3.31)	0.299
Control	1.65 (0.88 to 3.07)	0.117

Multivariable analyses were conducted using logistic regression, and results were expressed as OR and (95% CI) using the period before the COVID-19 pandemic as reference. Analyses were adjusted on gender, age (continuous), education (high, middle, low), marital status (living with partner, living alone), smoking (never, former, current), BMI categories (normal, overweight, obese).

BMI, body mass index.

### Association between SES and management of CVD risk factors before and during the COVID-19 pandemic

Table 4 depicts the prevalence, awareness, treatment and control rates of hypertension, diabetes, and dyslipidaemia before and during the pandemic according to educational level. Prevalence of hypertension was lower among high educated participants both before and during the pandemic. Awareness was similar before the pandemic but differed during the pandemic. Treatment rates were lower among high educated participants before the pandemic, but no difference was found during the pandemic. No differences regarding control rates were found between SES groups before and during the pandemic.

**Table 4:** Bivariate analysis of the association between educational level and management of cardiovascular risk factors before and during the COVID-19 pandemic, CoLaus study, Lausanne, Switzerland

		Before				During		
	High	Middle	Low	P value	High	Middle	Low	P value
Hypertension								
Prevalence	228 (43.7)	339 (48.3)	692 (58.1)	<0.001	90 (42.7)	98 (50.0)	216 (58.5)	<0.05
Awareness	173 (75.9)	271 (79.9)	565 (81.7)	0.165	67 (74.4)	59 (60.2)	173 (80.1)	<0.05
Treatment	135 (78.0)	238 (87.8)	488 (86.4)	<0.05	56 (83.6)	50 (84.8)	154 (89.0)	0.455
Control	82 (60.7)	146 (61.3)	291 (59.6)	0.900	41 (73.2)	28 (56.0)	92 (59.7)	0.130
Dyslipidaemia								
Prevalence	217 (41.6)	315 (44.9)	637 (53.4)	<0.001	76 (36.0)	77 (39.3)	175 (47.4)	<0.05
Awareness	134 (61.8)	192 (610)	406 (63.7)	0.676	45 (59.2)	48 (62.3)	116 (66.3)	0.541
Treatment	62 (46.3)	99 (51.6)	246 (60.6)	<0.05	19 (42.2)	23 (47.9)	73 (62.9)	0.032
Control	18 (29.0)	17 (17.2)	53 (21.5)	0.205	7 (36.8)	7 (30.4)	17 (23.3)	0.453
Diabetes								
Prevalence	32 (6.1)	54 (7.7)	154 (12.9)	<0.001	10 (4.7)	17 (8.7)	51 (13.8)	<0.05
Awareness	25 (78.1)	48 (88.9)	140 (90.9)	0.114	10 (100)	17 (100)	49 (96.1)	0.581§
Treatment	19 (76.0)	41 (85.4)	108 (77.1)	0.447	7 (70.0)	16 (94.1)	42 (85.7)	0.227
Control	6 (31.6)	12 (29.3)	44 (40.7)	0.379	3 (42.9)	8 (50.0)	21 (50.0)	0.938

Results are expressed as number of participants (percentage). Denominator for prevalence is total sample size: denominator for awareness is the no of participants with the condition (hypertension, diabetes or dyslipidaemia); denominator for treatment is the number of participants aware of the condition; denominator for control is the number of participants treated for the condition. Between educational groups comparisons performed using χ^2^ or Fisher’s exact test (§).

Prevalence of diabetes was lower among high educated participants both before and during the pandemic with no differences between SES groups regarding awareness, treatment and control rates before and during the pandemic (table 4).

Prevalence of dyslipidaemia was lower among high educated participants both before and during the pandemic. No differences were found between SES groups regarding awareness, while treatment rates were lower among high educated participants both before and during the pandemic. No differences were found between SES groups regarding control rates (table 4).

The results of the multivariable analysis of the association between SES and the management of CVD risk factors and the possible effect of the pandemic are summarised in table 5. Low educated participants tended to have a higher likelihood of hypertension compared with high educated participants with no effect of the pandemic. The likelihood of hypertension awareness decreased during the pandemic among middle educated participants (p for interaction<0.05). The likelihood of hypertension treatment was higher among middle educated participants before the pandemic and became non-significant during the pandemic, with no significant interaction with the pandemic period. No between SES differences were found regarding control of hypertension, and no changes occurred due to the pandemic (table 5).

**Table 5:** Multivariable analysis of the association between educational level and management of cardiovascular risk factors before and during the COVID-19 pandemic, CoLaus study, Lausanne, Switzerland

	Before				During				P for interaction	
	Middle versus high	P value	Low versus high	P value	Middle versus high	P value	Low versus high	P value	Middle	Low
Hypertension										
Prevalence	1.01 (0.78 to 1.31)	0.938	1.29 (1.01 to 1.64)	<0.05	1.30 (0.84 to 2.02)	0.245	1.40 (0.94 to 2.07)	0.097	0.371	0.768
Awareness	1.11 (0.73 to 1.69)	0.625	1.03 (0.70 to 1.50)	0.892	0.45 (0.23 to 0.85)	<0.05	1.10 (0.60 to 2.03)	0.748	<0.05	0.950
Treatment	1.81 (1.06 to 3.08)	<0.05	1.31 (0.83 to 2.08)	0.245	1.03 (0.34 to 3.12)	0.964	1.15 (0.43 to 3.06)	0.776	0.370	0.990
Control	1.03 (0.66 to 1.60)	0.898	0.93 (0.62 to 1.40)	0.743	0.46 (0.20 to 1.06)	0.067	0.54 (0.27 to 1.07)	0.077	0.054	0.120
Dyslipidaemia										
Prevalence	0.97 (0.77 to 1.23)	0.813	1.24 (1.00 to 1.55)	0.055	1.04 (0.69 to 1.59)	0.840	1.24 (0.86 to 1.79)	0.251	0.779	0.950
Awareness	1.01 (0.69 to 1.46)	0.977	1.05 (0.75 to 1.47)	0.795	0.89 (0.44 to 1.81)	0.754	1.11 (0.60 to 2.05)	0.750	0.982	0.584
Treatment	1.00 (0.60 to 1.67)	0.994	1.28 (0.81 to 2.03)	0.286	0.61 (0.21 to 1.77)	0.359	1.75 (0.68 to 4.49)	0.247	0.502	0.647
Control	0.36 (0.16 to 0.81)	<0.05	0.47 (0.23 to 0.94)	<0.05	0.92 (0.17 to 4.88)	0.925	0.48 (0.11 to 2.04)	0.323	0.324	0.860
Diabetes										
Prevalence	1.10 (0.68 to 1.77)	0.703	1.63 (1.07 to 2.49)	<0.05	1.5 (0.63 to 3.52)	0.357	1.98 (0.94 to 4.17)	0.073	0.437	0.366
Awareness	2.34 (0.65 to 8.46)	0.193	2.97 (0.97 to 9.15)	0.057	Not computable		Not computable			
Treatment	1.52 (0.42 to 5.42)	0.522	0.82 (0.28 to 2.42)	0.716	15.4 (0.76 to 314.5)	0.075	7.92 (0.73 to 85.4)	0.088	0.212	0.104
Control	0.94 (0.27 to 3.23)	0.923	1.38 (0.45 to 4.19)	0.574	2.18 (0.29 to 16.14)	0.446	1.99 (0.31 to 12.8)	0.470	0.607	0.856

Multivariable analyses were conducted using logistic regression, and results were expressed as OR and (95% CI). Analyses were adjusted on gender, age (continuous), marital status (living with partner, living alone), smoking (never, former, current) and BMI categories (normal, overweight, obese).

BMI, body mass index.

Low educated participants had a higher likelihood of diabetes compared with high educated participants, with no effect of the pandemic. No SES differences were found in awareness, treatment and control of diabetes, and the pandemic had no impact (table 5).

No between SES differences were found regarding prevalence, awareness, treatment and control of dyslipidaemia and pandemic had no impact (table 5). Similar findings were obtained when further adjusting for occupation (online supplemental tables 3 and 4)

## Discussion

This study showed that prevalence and management of CVD risk factors changed little during the pandemic, and that the SES gap did not increase, except for awareness of hypertension.

### Characteristics of the participants

Participants who attended the study centre during the pandemic were significantly younger than those who participated before the pandemic. The likely explanation is that on 13 March 2020, people aged 65 years and above were advised to avoid crowded places by the Swiss government as they were considered as vulnerable for the COVID-19 infection. Indeed, according to a recent study, over 90% of elderly people followed the recommendation in Lausanne.^18^

Participants attending during the pandemic were more frequently current smokers, a finding in agreement with studies conducted elsewhere.^19^ COVID-19 restrictive measures and social isolation negatively impacted the mental health of the Swiss population during the pandemic,^20^ which might be a reason for the increased prevalence of smokers during the pandemic. Conversely, a study conducted in the USA reported a decline in cigarette smoking, and suggested fear of increased COVID-19 risk and economic recession as possible explanations.^21^

The recruiting centre was close to the Lausanne University Hospital, which was the main hub for COVID-19 admissions. As overweight, obesity and diabetes were widely highlighted in the media as risk factors for severe COVID-19, it is likely that participants with those conditions tended to avoid coming to the recruiting centre.

Prevalence of alcohol consumption considerably declined from 70% before the pandemic to nearly half (30%) during the pandemic. This decrease is consistent with the findings in 21 European countries,^22^ and a likely explanation might be the closure of bars, pubs and other gather places imposed during the restrictive measures.^23^

### Prevalence and management of CVD risk factors

Prevalence of hypertension increased while awareness decreased during the pandemic. The increased prevalence is in line with a study in Spain,^24^ where mean SBP and DBP levels increased during lockdown. Those findings could partly be due to increased smoking,^21^ mental stress^20^ and salty food consumption during social isolation,^25^ which led to increased BP levels among participants who were not previously hypertensive. Overall, our results suggest that the lifestyle changes that occurred during the COVID-19 pandemic led to a significant increase in prevalence of hypertension, and possibly of new cases of hypertension.

The prevalence of dyslipidaemia declined during the pandemic and was further confirmed by multivariable analysis. This finding is in agreement with a study in Italy.^26^ The reasons for such a decline are unknown; possible explanations include the consumption of healthier foods^27^ although this statement has been challenged.^28^ In summary, our results suggest that prevalence of dyslipidaemia decreased during the COVID-19 pandemic in Switzerland, but the exact reasons remain to be assessed.

Prevalence of diabetes did not change, but the awareness increased substantially during the pandemic. A possible explanation is that people at risk of diabetes might have checked their status more frequently, as diabetes was considered as a major risk factor for increased risk of COVID-19-related mortality.^29^

The outbreak of the COVID-19 pandemic led to dramatic disruptions in CVD-related services in many countries.^11^ Restrictive and containment measures imposed as responses to the rapidly spreading pandemic also impacted in-person appointments, access to medicines and consequently treatment compliance in Switzerland.^30^ Nevertheless, it is possible that the decline of the in-person appointments in Switzerland during the pandemic might have been partly compensated by an increase in telemedicine. Indeed, telemedicine in Switzerland is relatively well developed and the use of digital services in healthcare was already increasing prior to the pandemic.^31^ Overall, our results suggest that the management of CVD risk factors in Switzerland changed little during the COVID-19 pandemic, and that a possible explanation would be the development of telemedicine, although this hypothesis remains to be confirmed.

### Association between educational level and management of CVD risk factors before and during the COVID-19 pandemic

There is little information regarding the effect of the COVID-19 pandemic on the SES gap of CVD risk factor management. In this study, we found few differences between educational groups regarding prevalence and management of CVD risk factors, and most of the existing differences did not widen during the pandemic. The sole exception was awareness of hypertension, which decreased among middle educated participants.

### Strengths and limitations

The major strengths of this study are that it is population based and was conducted using the same methodology before and during the pandemic.

This study also has several limitations. First, the study was conducted in a single location in Switzerland, a country with a resilient health system and where public health measures directed against COVID-19 differed from other countries. Hence, results might not be extrapolated to other settings. Second, we cannot rule out a possible selection bias; the participants who attended during the pandemic were younger, possessed a higher educational level, exhibited lower obesity rates, less consumed frequently alcohol and showed a slight increase in current smoking tendencies (table 1). Third, it was not possible to recruit during the first lock-down period, which might have precluded some participants to attend; similarly, it is likely that participants with comorbidities were less motivated to attend, as the risk of developing severe COVID-19 was higher. Hence, it is possible that awareness, treatment and control rates be overestimated. Still, no such overestimation was observed relative to the prepandemic period and after the lockdown measures were lifted, recruitment resumed as previously. Finally, the sample size collected during the pandemic was relatively small, thus limiting statistical power. Still, to our knowledge, this is the sole study conducted in a population setting in Switzerland during the COVID-19 pandemic.

### Conclusion

We conclude that the prevalence and management of CVD risk factors changed little during the pandemic. The SES gap did not increase except for hypertension awareness.

## Data Availability

Data are available on reasonable request. The data of CoLaus|PsyCoLaus study used in this article cannot be fully shared as they contain potentially sensitive personal information on participants. According to the Ethics Committee for Research of the Canton of Vaud, sharing these data would be a violation of the Swiss legislation with respect to privacy protection. However, coded individual-level data that do not allow researchers to identify participants are available upon request to researchers who meet the criteria for data sharing of the CoLaus|PsyCoLaus Datacenter (CHUV, Lausanne, Switzerland). Any researcher affiliated to a public or private research institution who complies with the CoLaus|PsyCoLaus standards can submit a research application to research.colaus@chuv.ch or research.psycolaus@chuv.ch. Proposals requiring baseline data only, will be evaluated by the baseline (local) Scientific Committee (SC) of the CoLaus and PsyCoLaus studies. Proposals requiring follow-up data will be evaluated by the follow-up (multicentric) SC of the CoLaus|PsyCoLaus cohort study. Detailed instructions for gaining access to the CoLaus|PsyCoLaus data used in this study are available at www.colaus-psycolaus.ch/professionals/how-to-collaborate/.
