# Supplementary figures and images for: Impact of the COVID-19 pandemic on CVD prevention between different socioeconomic groups in Switzerland
Source: Open Heart. 2023 Sep 19;10(2):e002368. doi: 10.1136/openhrt-2023-002368 (PMC10510922; doi:10.1136/openhrt-2023-002368)

## Supplementary figure 1: selection procedure

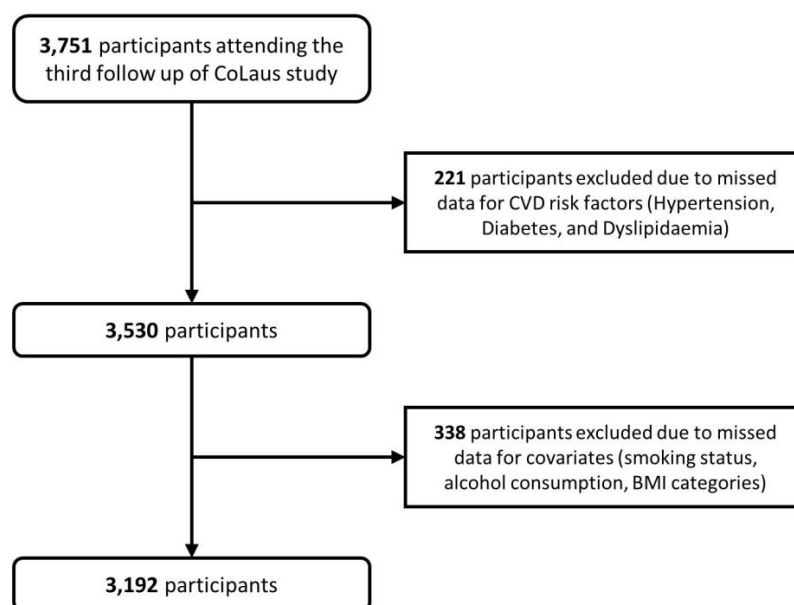

Supplement: Supplementary data [file openhrt-2023-002368supp001.pdf]
