# Supplementary material for: Impact of the COVID-19 pandemic on CVD prevention between different socioeconomic groups in Switzerland
Source: Open Heart. 2023 Sep 19;10(2):e002368. doi: 10.1136/openhrt-2023-002368 (PMC10510922; doi:10.1136/openhrt-2023-002368)
Supplement: Supplementary data [file openhrt-2023-002368supp002.pdf]

SUPPLEMENTARY TABLES

Supplementary Table 1: Bivariate analysis between excluded and included participants

	Included	Excluded	P-value
**Sample size**	**3,912**	**559**	
Women (%)	1,755 (55.0)	329 (58.9)	0.089
Age (years)	65.0 ± 9.7	66.8 ± 10.9	<0.001
Swiss born (%)	2,080 (65.2)	296 (53.0)	<0.001
Education (%)			
Low	733 (23.0)	113 (20.3)	<0.001
Middle	898 (28.1)	127 (22.8)	
High	1,561 (48.9)	317 (56.9)	
Smoking categories (%)			
Never	1,408 (44.1)	87 (42.9)	<0.001
Former	1,276 (40.0)	80 (39.4)	
Current	508 (15.9)	36 (17.7)	
Body mass index (kg/m^2)	26.3 ± 4.6	26.9 ± 5.0	<0.05
BMI categories (%)			
Normal	1,364 (42.7)	137 (38.4)	<0.001
Overweight	1,223 (38.3)	133 (37.3)	
Obese	605 (19.0)	87 (24.4)	
History of CVD (%)			
Personal	425 (13.3)	109 (19.5)	<0.001
Family	2,270 (71.1)	333 (59.6)	<0.001
Alcohol drinker (%)	1,989 (62.3)	70 (12.5)	<0.001

Results are expressed as number of participants (percentage) for categorical variables and as average±standard deviation for continuous variables. Between-group comparisons performed using chi-square for categorical variables and student's t-test for continuous variables.

Maung KK, Marques-Vidal P. *Open Heart* 2023; 10:e002368. doi: 10.1136/openhrt-2023-002368

Supplementary table 2: Bivariate analysis of the management of cardiovascular risk factors between men and women before and during the COVID-19 pandemic, CoLaus study, Lausanne, Switzerland.

	Before		During		P-value
	Men	Women	Men	Women	
**Hypertension (%)**					
Prevalence	644 (51.2)	615 (48.9)	209 (51.7)	195 (48.3)	0.839
Awareness	510 (50.6)	499 (49.5)	149 (49.8)	150 (50.2)	0.829
Treatment	429 (49.8)	432 (50.2)	131 (50.4)	129 (49.6)	0.875
Control	251 (48.4)	268 (51.6)	79 (49.1)	82 (50.9)	0.876
**Dyslipidaemia (%)**					
Prevalence	494 (42.3)	675 (57.7)	146 (44.5)	182 (55.5)	0.466
Awareness	343 (46.9)	389 (53.1)	108 (51.7)	101 (48.3)	0.219
Treatment	213 (52.3)	194 (47.7)	63 (54.8)	52 (45.2)	0.642
Control	34 (38.6)	54 (61.4)	13 (41.9)	18 (58.1)	0.747
**Diabetes (%)**					
Prevalence	147 (61.3)	93 (38.8)	41 (52.6)	37 (47.4)	0.175
Awareness	125 (58.7)	88 (41.3)	41 (54.0)	35 (46.1)	0.473
Treatment	95 (56.6)	73 (43.5)	36 (55.4)	29 (44.6)	0.872
Control	31 (50.0)	31 (50.0)	19 (59.4)	13 (40.6)	0.388

Results are expressed as number of participants (percentage). Denominator for prevalence is total sample size; denominator for awareness is the number of participants with the condition (hypertension, diabetes, or dyslipidaemia); denominator for treatment is the number of participants aware of the condition; denominator for control is the number of participants treated for the condition. Between-group comparisons performed using chi-square.

Maung KK, Marques-Vidal P. *Open Heart* 2023; 10:e002368. doi: 10.1136/openhrt-2023-002368

Supplementary table 3: Multivariable analysis of the management of cardiovascular risk factors during the COVID-19 pandemic, CoLaus study, Lausanne, Switzerland.

	**During the pandemic**	**P-value**
**Hypertension**		
Prevalence	1.27 (1.05 - 1.54)	0.016
Awareness	0.70 (0.52 - 0.93)	0.015
Treatment	1.32 (0.87 – 2.00)	0.198
Control	1.07 (0.78 - 1.45)	0.685
**Dyslipidaemia**		
Prevalence	0.82 (0.69 - 0.98)	0.033
Awareness	1.06 (0.81 - 1.40)	0.657
Treatment	0.92 (0.63 - 1.34)	0.675
Control	1.55 (0.91 - 2.66)	0.108
**Diabetes**		
Prevalence	0.93 (0.69 - 1.26)	0.633
Awareness	5.82 (1.24 - 27.3)	0.026
Treatment	1.57 (0.71 - 3.46)	0.265
Control	1.61 (0.86 - 3.02)	0.134

Multivariable analyses were conducted using logistic regression, and results were expressed as odds ratio and (95% confidence interval) using the period before the COVID-19 pandemic as reference. Analyses were adjusted on gender, age (continuous), education (high, middle, low), marital status (living with partner, living alone), smoking (never, former, current), BMI categories (normal, overweight, obese), and having an occupation (yes, no)

Supplementary table 4: Multivariable analysis of the association between occupational status and management of cardiovascular risk factors before and during the COVID-19 pandemic, CoLaus study, Lausanne, Switzerland.

	Before		During		P for interaction
	**Occupation (yes vs no)**	**P value**	**Occupation (yes vs no)**	**P value**	**Occupation (yes)**
**Hypertension**					
Prevalence	1.17 (0.91 - 1.51)	0.219	0.90 (0.59 - 1.37)	0.623	0.438
Awareness	1.27 (0.85 - 1.90)	0.241	0.75 (0.41 - 1.37)	0.350	0.609
Treatment	1.12 (0.68 - 1.85)	0.659	0.34 (0.13 - 0.92)	0.033	0.002
Control	1.13 (0.76 - 1.68)	0.551	1.52 (0.78 - 2.94)	0.217	0.824
**Dyslipidaemia**					
Prevalence	0.68 (0.54 - 0.85)	0.001	1.14 (0.77 - 1.69)	0.514	0.061
Awareness	1.05 (0.34 - 3.31)	0.927	0.73 (0.39 - 1.38)	0.337	0.205
Treatment	1.13 (0.71 - 1.79)	0.603	0.74 (0.32 - 1.70)	0.476	0.080
Control	0.95 (0.45 - 1.97)	0.884	1.12 (0.30 - 4.23)	0.862	0.609
**Diabetes**					
Prevalence	0.91 (0.61 - 1.36)	0.648	0.39 (0.20 - 0.77)	0.007	0.011
Awareness	1.05 (0.34 - 3.31)	0.927	Not computable		Not computable
Treatment	0.82 (0.32 - 2.11)	0.682	11.2 (0.78 - 159.7)	0.075	0.103
Control	0.63 (0.25 - 1.57)	0.318	0.42 (0.11 - 1.61)	0.204	0.875

Multivariable analyses were conducted using logistic regression, and results were expressed as odds ratio and (95% confidence interval). Analyses were adjusted on gender, age (continuous), marital status (living with partner, living alone), smoking (never, former, current), and BMI categories (normal, overweight, obese)

Maung KK, Marques-Vidal P. *Open Heart* 2023; 10:e002368. doi: 10.1136/openhrt-2023-002368
